# A role for IL-33–activated ILC2s in eosinophilic vasculitis

**DOI:** 10.1172/jci.insight.143366

**Published:** 2021-06-22

**Authors:** Maya E. Kotas, Jérémie Dion, Steven Van Dyken, Roberto R. Ricardo-Gonzalez, Claire J. Danel, Camille Taillé, Luc Mouthon, Richard M. Locksley, Benjamin Terrier

**Affiliations:** 1 Division of Pulmonary, Critical Care, Allergy & Sleep Medicine, University of California, San Francisco, California, USA.; 2 Department of Internal Medicine, National Referral Center for Rare and Systemic Autoimmune Diseases, Cochin Hospital, AP-HP, Paris, France.; 3 Department of Pathology & Immunology, Washington University School of Medicine in St. Louis, Missouri, USA.; 4 Department of Dermatology, University of California, San Francisco, California, USA.; 5 Department of Pathology and; 6 Department of Pulmonology, Bichat Hospital, AP-HP, Paris, France.; 7 Howard Hughes Medical Institute, University of California, San Francisco, California, USA.; 8 Department of Medicine, University of California, San Francisco, California, USA.

**Keywords:** Autoimmunity, Immunology, Mouse models, Rheumatology, Vasculitis

## Abstract

Eosinophilic granulomatosis with polyangiitis (EGPA) is a rare but serious disease with poorly understood mechanisms. Here, we report that patients with EGPA have elevated levels of TSLP, IL-25, and soluble ST2, which are well-characterized cytokine “alarmins” that activate or modulate type 2 innate lymphoid cells (ILC2s). Patients with active EGPA have a concurrent reduction in circulating ILC2s, suggesting a role for ILC2s in the pathogenesis of this disease. To explore the mechanism of these findings in patients, we established a model of EGPA in which active vasculitis and pulmonary hemorrhage were induced by IL-33 administration in predisposed, hypereosinophilic mice. In this model, induction of pulmonary hemorrhage and vasculitis was dependent on ILC2s and signaling through IL4Rα. In the absence of IL4Rα or STAT6, IL-33–treated mice had less vascular leak and pulmonary edema, less endothelial activation, and reduced eotaxin production, cumulatively leading to a reduction of pathologic eosinophil migration into the lung parenchyma. These results offer a mouse model for use in future mechanistic studies of EGPA, and they suggest that IL-33, ILC2s, and IL4Rα signaling may be potential targets for further study and therapeutic targeting in patients with EGPA.

## Introduction

Eosinophilic granulomatosis with polyangiitis (EGPA), is a rare but potentially life-threatening disease with few effective treatments (1–3). The initial phase, characterized by asthma and other allergic symptoms, is followed by tissue and blood hypereosinophilia, and later by systemic necrotizing vasculitis (1–3). Eosinophils, which typically represent approximately 1% of peripheral blood leukocytes (4), reach 10% or greater in EGPA (3). Eosinophils accumulate in the extravascular space, sometimes in pathognomonic granulomas. Their aggregation and activation around small vessels result in ischemic or thrombotic tissue damage (“vasculitis”) (3). Accordingly, treatment with anti–IL-5 mAb yields clinical improvement in some patients (2). Hypereosinophilia alone — which also occurs in medication reactions, hematologic malignancy, allergy, or parasite infection — is not sufficient to cause vasculitis (5–8). But it remains unclear what additional signals induce disease, or what tissue specific signals explain predilection for particular organs such as airways, peripheral nerves, skin, and heart (3).

Given the similarities with allergic asthma, the presumption has been that eosinophil activity is driven by Th type 2 (Th2) cells. However, this hypothesis remains unproven, in part because rodent models that recapitulate human disease are lacking (3). In addition, a role for type 2 innate lymphoid cells (ILC2s), tissue-resident innate counterparts to Th2 cells that are highly responsive to IL-25, TSLP, and IL-33 (9), has not been explored. ILC2s constitutively produce IL-5, which is important for controlling tissue accumulation of eosinophils (10) as well as other type 2 cytokines such as IL-4 and IL-13. In this study, we examine a role for tissue cytokines in activation of ILC2s in patients with EGPA and establish a mouse model to study this complex and devastating disease.

## Results

### Patients with EGPA had elevated type 2 cytokines in serum and altered ILC2 numbers.

**Figure 1:**
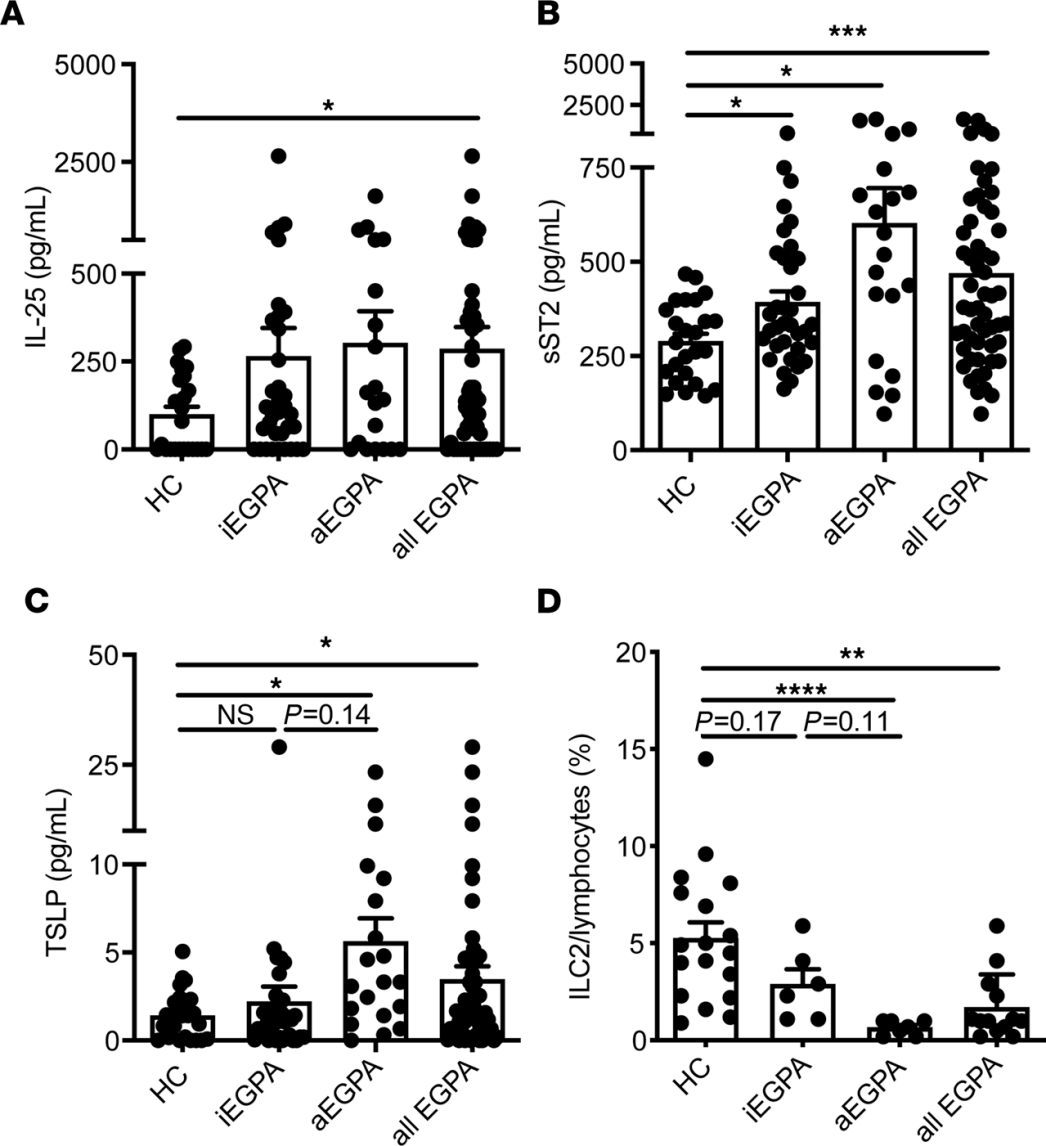
Patients with EGPA have elevated type 2-activating cytokines and altered ILC2 numbers in blood. (**A**) IL-25, (**B**) sST2, and (**C**) TSLP from HCs or patients with EGPA with iEGPA or aEGPA. *n* = 20–53 per group. (**D**) Peripheral blood ILC2s measured from HCs or patients with EGPA with iEGPA or aEGPA. ****P < 0.0001; ***P < 0.001; **P < 0.01; *P < 0.05 by Brown-Forsythe and Welch ANOVA with Dunnett’s post hoc testing. *n* = 6–18 patients per group. EGPA, eosinophilic granulomatosis with polyangiitis; ILC2s, type 2 innate lymphoid cells; sST2, soluble ST2; HCs, healthy controls; iEGPA, inactive disease; aEGPA, active disease.

We quantified increased serum levels of IL-5 and IL-10 among patients with EGPA (Supplemental Figure 1, A and B; supplemental material available online with this article; https://doi.org/10.1172/jci.insight.143366DS1), particularly during active disease. We reasoned that alarmins known to potentiate IL-5 production might act upstream of these effector cytokines in patients with EGPA. GWAS analysis identifies variants in the gene encoding TSLP in patients with EGPA (11), and both IL-25 (12) and IL-33 (13) have previously been reported to be elevated in patients with active EGPA. Indeed, we observed that patients with EGPA had elevated serum levels of IL-25, TSLP, and soluble ST2 (sST2), the decoy receptor for IL-33 (Figure 1, A–C), whereas IL-33 was not detected in serum from patients with EGPA or controls (data not shown).

Because these cytokines can activate ILC2s, a major source of IL-5, we hypothesized that ILC2s might contribute to disease in patients with EGPA. Peripheral blood ILC2s were significantly decreased in patients with EGPA (Figure 1D and Supplemental Figure 1C). Because ILC2s are predominantly tissue resident (14), we hypothesize that elevated tissue cytokines may induce migration and retention of circulating ILC2s into diseased tissues in EGPA, as previously described in allergic asthma (15).

### IL-33 treatment in hypereosinophilic mice caused eosinophilic vasculitis.

To test the role of ILC2s and their activating cytokines in pathogenesis of eosinophilic vasculitis, we employed a previously described mouse model wherein IL-5 is expressed as a transgene from the CD3δ promoter (herein designated as “IL5Tg”), resulting in approximately 100-fold normal levels of circulating eosinophils (>60% of total peripheral blood leukocytes, progressive with age) (5). These mice have diffuse organ infiltration with eosinophils, splenomegaly, and lymphocytosis but do not exhibit spontaneous eosinophilic vasculitis.

**Figure 2:**
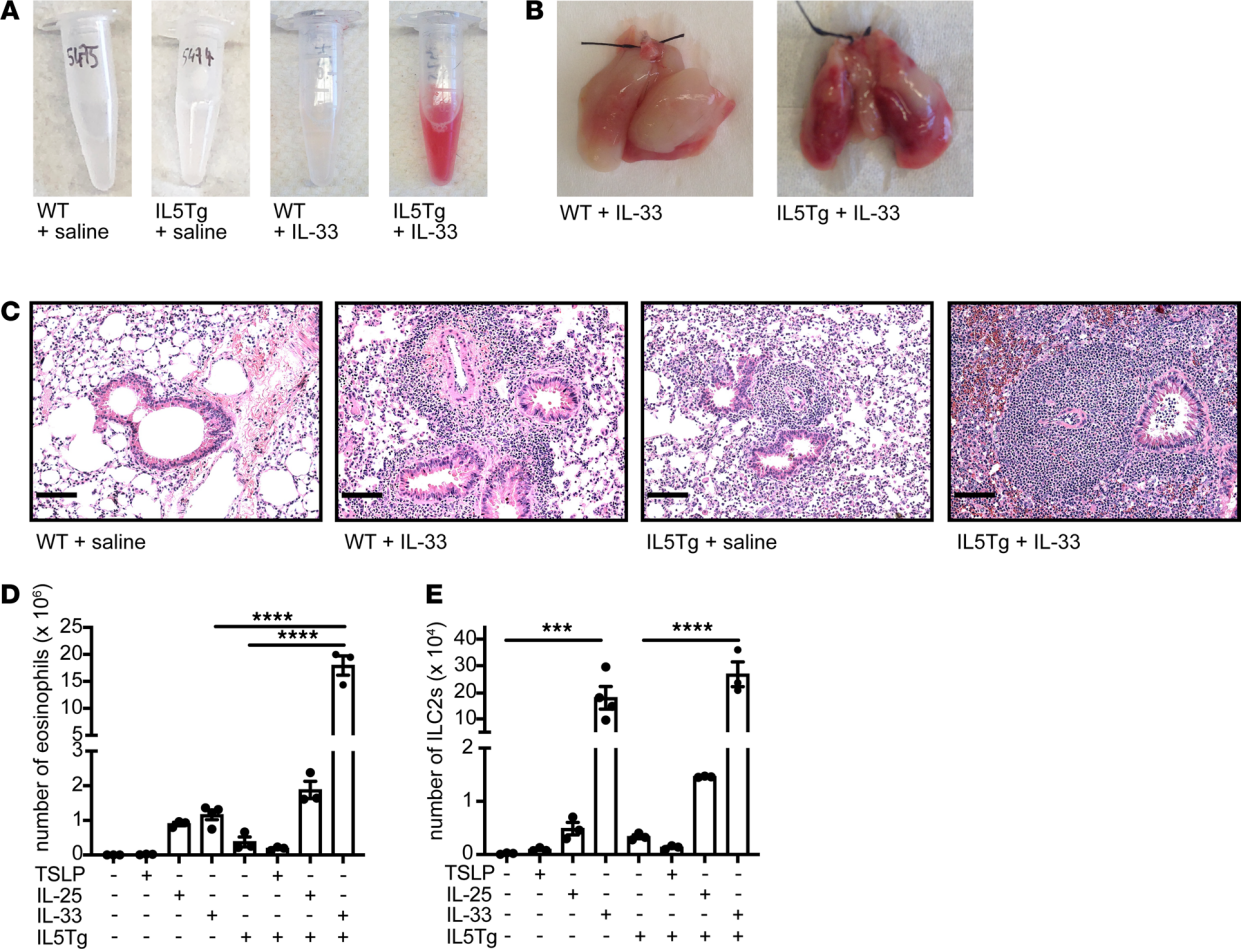
IL-33 and hypereosinophilia synergize to cause eosinophilic vasculitis in mice. (**A**) Representative bronchoalveolar lavage and (**B**) representative whole lungs from IL-33–treated WT or IL5Tg mice. Images in **A** and **B** are representative of 3 or more mice/group, in a minimum of 3 independent experiments. (**C**) H&E staining of lungs from representative IL-33–treated WT or IL5Tg mice, as shown in **A** and **B** (original magnification, ×20). Scale bars: 100 μm. (**D**) Eosinophils or (**E**) ILC2s in BAL. Data are presented as ± SEM. ****P < 0.0001; ***P < 0.001 by 1-way ANOVA with Sidak post hoc testing. *n* = 3–4 mice/group. BAL, bronchoalveolar lavage; ILC2s, type 2 innate lymphoid cells.

Treatment of IL5Tg mice intranasally with IL-33 but not IL-25, TSLP, or saline over a 2-week period led to alveolar hemorrhage (Figure 2, A and B, and Supplemental Figure 3A). Saline-treated IL5Tg mice showed pulmonary eosinophilia with peribronchovascular eosinophilic clusters (Figure 2C), as previously described (5, 8). IL-33–treated WT mice showed lymphocytic infiltration and perivascular lymphoid aggregates, consistent with lymphocytic pneumonia (Figure 2C). In contrast, lungs of IL-33–treated IL5Tg mice showed dense patches of perivascular and peribronchial inflammatory infiltrates, individual and clustered multinucleated giant cells, nodular areas of necrosis, intra-alveolar fibrin deposits, eosinophilic vasculitis, and alveolar hemorrhage (Figure 2C and Supplemental Figure 3, B–G). Treatment of IL5Tg mice with IL-33, but not IL-25 or TSLP, also induced a synergistic increase in bronchoalveolar lavage (BAL) eosinophilia (Figure 2D and Supplemental Figure 2). IL-33 dramatically increased ILC2s in the BAL in both WT and IL5Tg mice (Figure 2E), as previously described (16). The number of eosinophils and ILC2s in lung tissue and BAL were closely correlated, indicating the utility of BAL in assessing the tissue inflammatory state (Supplemental Figure 3, H and I).

EGPA can manifest in numerous tissues, including skin. Although we observed collections of eosinophils in skin of saline-treated IL5Tg mice, the cutaneous vessels appeared normal (Supplemental Figure 3J). However, when IL5Tg mice were treated with IL-33, we observed eosinophilic infiltration of the perivascular space and vascular walls (Supplemental Figure 3J). Antineutrophil cytoplasm Abs (ANCAs), which are found in about a third of patients with EGPA (17), were not increased in IL5Tg mice, with or without cytokine treatment (data not shown). Thus, IL-33 treatment of IL5Tg mice recapitulated aspects of eosinophilic vasculitis in patients with EGPA.

### ILC2s were required for the development of vasculitis in hypereosinophilic mice.

**Figure 3:**
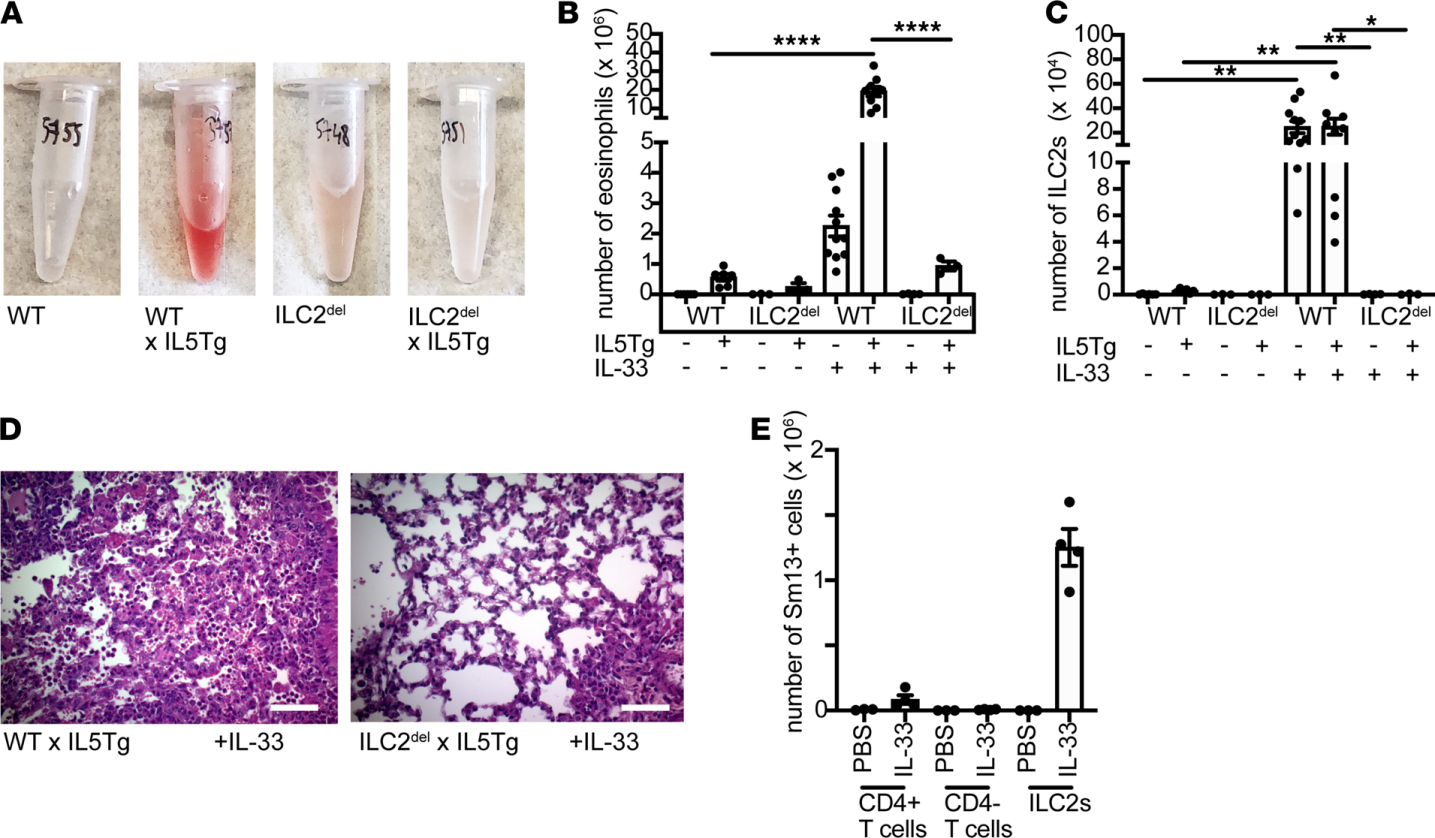
ILC2s are required for development of vasculitis in hypereosinophilic mice. (**A**) BAL from ILC2-deficient ILC2^del^ x IL5Tg mice (representative of 3 mice/group). (**B**) Eosinophils or (**C**) ILC2s in BAL of IL-33–treated ILC2^del^ x IL5Tg. ****P < 0.0001, **P < 0.01, *P < 0.05 by 1-way ANOVA with Sidak post hoc testing. *n* = 3–11 mice/group. (**D**) H&E of lungs from representative IL-33–treated IL5Tg or ILC2^del^ x IL5Tg mice treated with IL-33 shown in **A**–**C** (original magnification, ×40). Scale bars: 100 μm. (**E**) Number of IL-13^Sm13+^ cells in lungs of saline-treated or IL-33–treated YYRS Smart13–knock-in mice expressing a modified human CD4 marker from the mouse IL-13 gene locus. ILC2 defined as lin^–^, Thy1.2^+^, CD3^–^, Arg1^YFP+^, Red5^+^. Data are presented as ± SEM. *n* = 3–4 mice/group. BAL, bronchoalveolar lavage; ILC2s, type 2 innate lymphoid cells.

To test the role of ILC2s in vasculitis, we crossed IL5Tg mice to *Il5*^Red5Cre^ROSA*-*DTA ILC2-deficient R5/R5 deleter (ILC2^del^) mice, in which mature ILC2s — which constitutively produce IL-5 — are deleted via Cre-inducible expression of diphtheria toxin alpha from the endogenous IL-5 promoter (10). ILC2^del^ x IL5Tg mice were protected from pulmonary hemorrhage after IL-33 (Figure 3A) and had dramatically blunted BAL eosinophilia (Figure 3B) despite insignificant changes in peripheral eosinophilia (Supplemental Figure 4B). As expected, ILC2s were absent from the BAL in ILC2^del^ mice (Figure 3C). ILC2^del^ mice were rescued from histopathologic findings of eosinophilic vasculitis (Figure 3D). Even during IL-33 administration, ILC2s were the predominant population of IL-5–expressing cells in the mouse lung (Supplemental Figure 4A), indicating that deletion of Th2 cells was unlikely to explain the absence of vasculitis.

### IL4Rα signaling was required for the development of vasculitis in hypereosinophilic mice.

ILC2s produce numerous effector molecules, including IL-5, IL-13, IL-9, and amphiregulin (9). We reasoned that in the context of systemic IL-5 overexpression, the contribution of ILC2-derived IL-5 was likely negligible. After IL-33 administration, ILC2s were the major IL-13–producing cell type in the lung (Figure 3E), suggesting that ILC2-derived IL-13 might have been implicated in the pathogenesis of eosinophilic vasculitis in our model.

**Figure 4:**
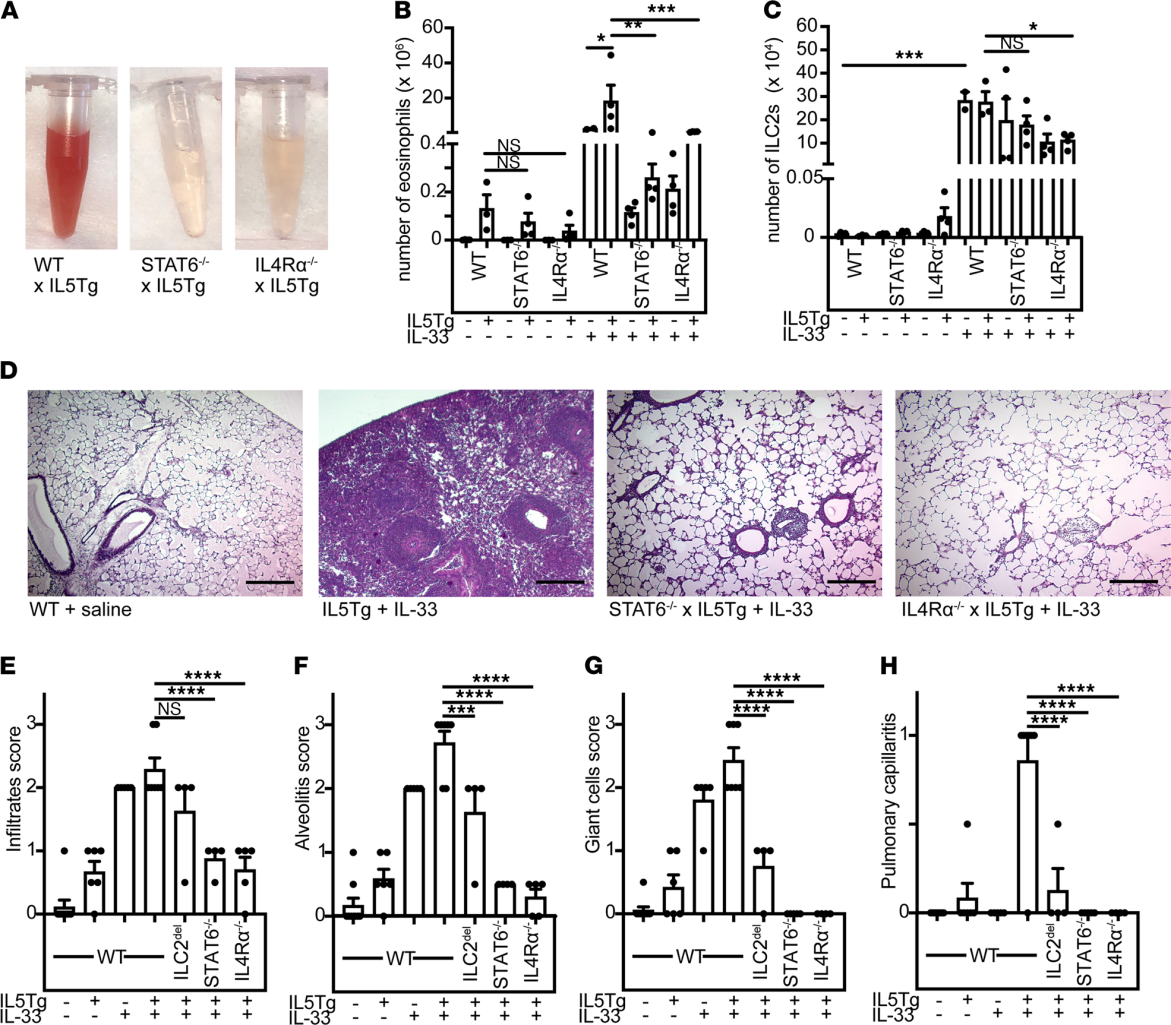
Deficiency of IL4Rα signaling protects from vasculitis in hypereosinophilic mice. (**A**) BAL from IL-33–treated IL5Tg, STAT6^–/–^ x IL5Tg, or IL4Rα^–/–^ x IL5Tg. Representative of 4 or more mice/group. (**B**) Eosinophils in BAL of mice from **A**. Data are presented as ± SEM. ****P < 0.0001; **P < 0.01; *P < 0.05; ns = by 1-way ANOVA with Sidak post hoc testing. *n* = 2–4 mice/group. (**C**) ILC2s in BAL of mice from **A**. ***P < 0.001; *P < 0.05; ns = by 1-way ANOVA with Sidak post hoc testing. *n* = 2–4 mice/group. (**D**) H&E staining of lungs from representative IL-33–treated IL5Tg, STAT6^–/–^ x IL5Tg or IL4Rα^–/–^ x IL5Tg mice treated with IL-33, as shown in **A**–**C** (original magnification, ×10). Scale bars: 100 μm. (**E**) Histopathological scoring for infiltrates, (**F**) alveolitis, (**G**) giant cells, and (**H**) pulmonary capillaritis in ILC2^del^, STAT6^–/–^, or IL4Rα^–/–^ with or without IL5Tg after saline or IL-33 treatment. ****P < 0.0001, ***P < 0.001, **P < 0.01; *P < 0.05 by 1-way ANOVA with Dunnett’s post hoc testing. *n* = 4–9 mice/group. Data are presented as ± SEM. BAL, bronchoalveolar lavage; ILC2s, type 2 innate lymphoid cells.

To investigate the role of IL4Rα signaling in our model, we generated IL5Tg mice with cells unresponsive to IL-4 and IL-13 signaling by crossing to IL4Rα^–/–^ (the receptor for IL-4 and IL-13) or STAT6^–/–^ (required for downstream signaling). Like ILC2^del^ mice, IL4Rα^–/–^ x IL5Tg and STAT6-^–/–^ x IL5Tg mice were protected from pulmonary hemorrhage after IL-33 administration (Figure 4A) and had dramatically reduced BAL eosinophilia compared with controls (Figure 4B). The number of ILC2s in the BAL, however, was unaffected in STAT6^–/–^ mice and minimally affected by the loss of IL4Rα (Figure 4C). Thus, IL-4 and IL-13 signaling is dispensable for ILC2 expansion and appearance in the BAL and may rather be necessary for their pathologic effect. Consistent with the loss of hemorrhage and eosinophils from BAL, IL5Tg mice on either IL4Rα^–/–^ or STAT6^–/–^ backgrounds were rescued from histopathologic evidence of eosinophilic vasculitis (Figure 4, D–H). The degree of rescue was more profound with STAT6^–/–^ or IL4Rα^–/–^ than with ILC2^del^ (Figure 4, E–H), suggesting either incomplete deletion of ILC2s or production of IL-13 or IL-4 by other cells. Importantly, STAT6^–/–^ x IL5Tg or IL4Rα^–/–^ x IL5Tg mice had comparable blood eosinophilia, and ILC2 expansion after IL-33 treatment was equivalent (Supplemental Figure 5, A and B).

### IL-33 caused IL4Rα-dependent acute lung injury and pulmonary vascular leak.

**Figure 5:**
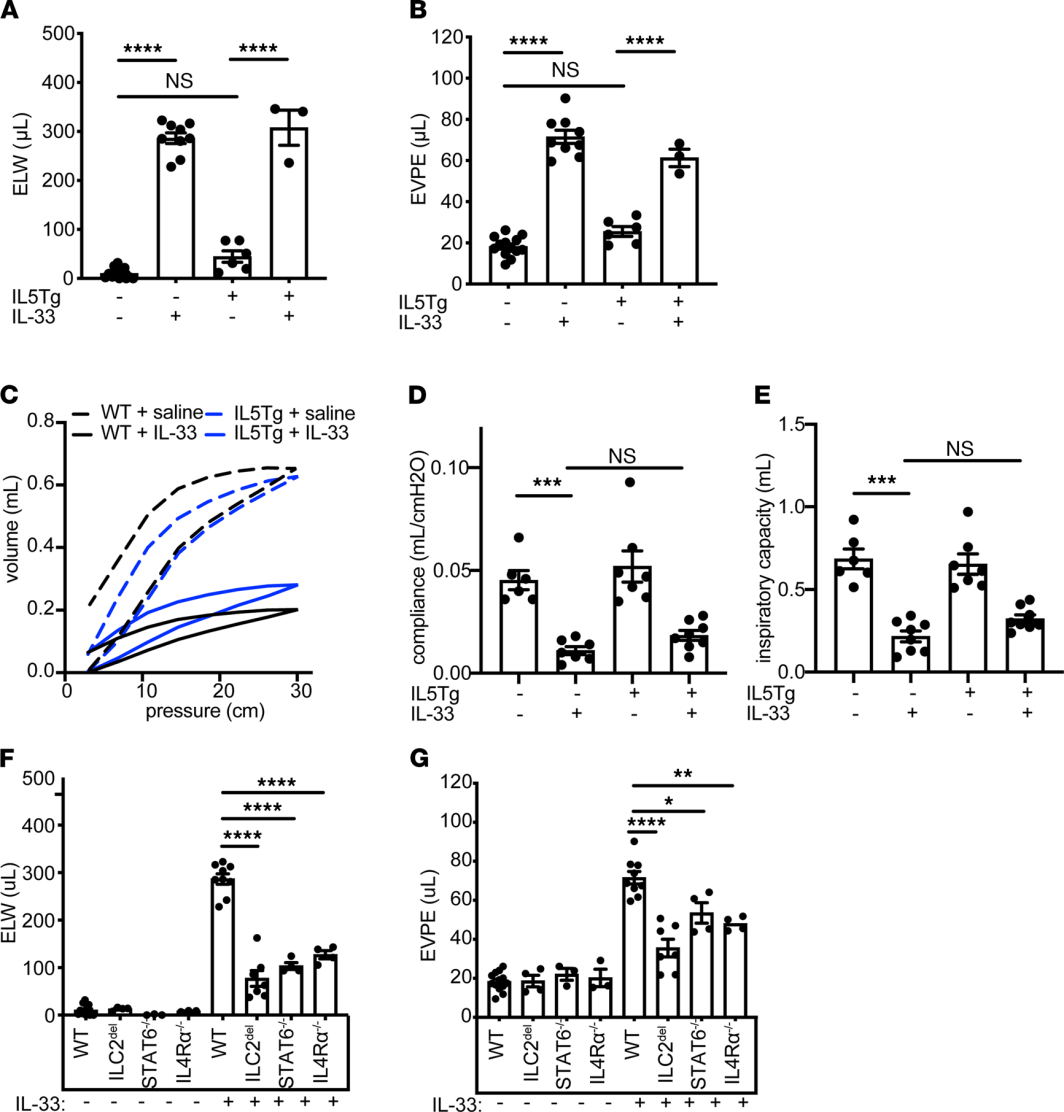
IL-33 induces IL4Rα-dependent vascular leak, resulting in pulmonary edema. (**A**) ELW of saline-treated or IL-33–treated WT or IL5Tg mice. *n* = 3–13 mice/group. (**B**) EVPE measured from saline-treated or IL-33–treated WT or IL5Tg mice. *n* = 3–13 mice/group. (**C**) Pressure volume curves, (**D**) lung static compliance, and (**E**) lung inspiratory capacity in indicated mice. *n* = 6–8 mice/group. (**F**) ELW or (**G**) EVPE in genotypes as indicated. *n* = 3–9 mice/group. Significance determined by 1-way ANOVA with Sidak post hoc testing (**A**, **B**, **F**, and **G**), or Brown-Forsyth and Welch ANOVA with Dunnett’s multiple comparisons test (**D** and **E**). ****P < 0.0001; ***P < 0.001; **P < 0.01; *P < 0.05. Data are presented as ± SEM. EVPE, extravascular plasma equivalents; ELW, excess/extravascular lung water.

We suspected that IL-33-induced IL4Rα/STAT6-dependent signal(s) give eosinophils access to tissue-derived factors, resulting in degranulation and pathology. Indeed, IL-33 treatment caused severe pulmonary edema, as measured by excess/extravascular lung water (ELW), and increased vascular permeability, as measured by extravascular plasma equivalents (EVPE; Figure 5, A and B). In contrast, TSLP did not increase ELW or EVPE, and IL-25 caused substantially less pulmonary edema than IL-33 (Supplemental Figure 6, A and B).

Pulmonary edema resulted in a loss of static compliance (Figure 5, C and D) and reduced inspiratory capacity indicative of a restrictive physiology (Figure 5E) that was unaffected by the IL5Tg. We did not observe airway hyperreactivity suggestive of asthma (Supplemental Figure 6, C–F), suggesting this model more accurately reflects the active vasculitis phase of EGPA than the asthmatic, prodromal phase.

We hypothesized that IL-33 treatment may act through ILC2s to stimulate IL-13 production, leading to acute lung injury, vascular permeability, and resulting pulmonary edema. Consistent with this hypothesis, we found that ILC2-deficient or IL-4 and IL-13 signaling–deficient mice had significantly reduced ELW and EVPE after IL-33 compared with WT mice (Figure 5, F and G). Comparatively, Rag1^–/–^ mice were not protected from increased ELW and EVPE after IL-33, suggesting that the IL-4 and IL-13 signaling that induced vascular permeability and pulmonary edema was not dependent on T cells (Supplemental Figure 6, G and H).

### Signaling through IL4Rα-mediated movement of eosinophils from the vasculature to the extravascular space.

**Figure 6:**
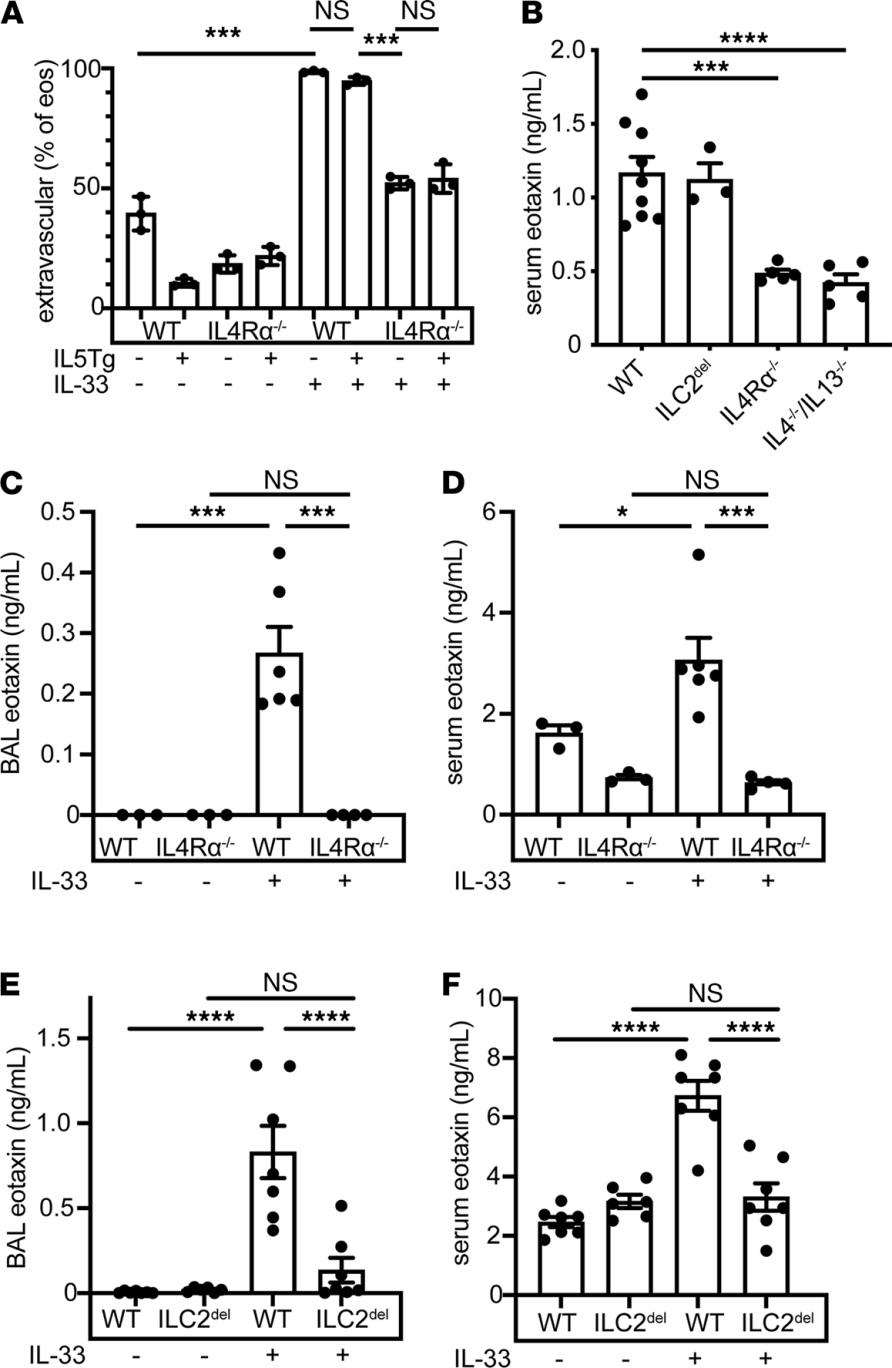
IL4Rα signaling facilitates eosinophil egress into tissue through recruitment and vascular activation. (**A**) Extravascular (unlabeled) eosinophils (single, live, CD45^+^, CD11b^+^, CD125^–^, SiglecF^+^, SSC^hi^ cells) from saline-treated or IL-33–treated lungs. *n* = 3 mice/group. (**B**) Eotaxin measured from serum of untreated mice as indicated. *n* = 3–9 mice/group. (**C**) Eotaxin measured from BAL or (**D**) serum of saline-treated or IL-33–treated WT or IL4Rα^–/–^ mice. *n* = 3–6 mice/group. (**E**) Eotaxin measured from BAL or (**F**) serum of saline-treated or IL-33–treated WT or ILC2^del^ mice. *n* = 6–7 mice/group. Significance determined by 1-way ANOVA with Sidak (**A**, **C**, and **D**) Dunnett’s (**B**) or Tukey (**E** and **F**) post hoc testing; ****P < 0.0001; ***P < 0.001; *P < 0.05. Data are presented as ± SEM. BAL, bronchoalveolar lavage; ILC2s, type 2 innate lymphoid cells.

Because IL5Tg mice on ILC2^del^, STAT6^–/–^ or IL4Rα^–/–^ backgrounds had severe hypereosinophilia but few pulmonary intraparenchymal eosinophils and no vasculitis, we suspected that IL4Rα signaling might promote eosinophil migration into tissue. Few lung eosinophils from saline-treated WT or IL5Tg mice remained unlabeled after intravenous injection of anti-CD45 Ab, consistent with predominantly intravascular localization (Figure 6A). After IL-33 treatment, however, more than 90% of the pulmonary eosinophils were unlabeled, consistent with tissue entry (Figure 6A and Supplemental Figure 7A). By comparison, only approximately 50% as many eosinophils reached the extravascular space in IL-33–treated IL4Rα^–/–^ or IL4Rα^–/–^ x IL5Tg mice (Figure 6A and Supplemental Figure 7B).

Signaling via IL4Rα is known to stimulate the production of the chemokine eotaxin by multiple lung cell types in patients with asthma (18). Consistent with these clinical observations, we found that both IL4Rα^–/–^ mice and IL4^–/–^/IL13^–/–^ mice had significantly decreased levels of eotaxin in serum in the unperturbed state (Figure 6B). Further, eotaxin levels in both BAL and serum were strongly induced after IL-33 treatment in WT mice but not in IL4Rα^–/–^ mice (Figure 6, C and D). Although ILC2^del^ mice did not have decreased serum eotaxin at baseline, we reasoned that in the setting of IL-33–induced ILC2 activation and IL-13 production, eotaxin levels may depend on ILC2s. Indeed, eotaxin was strongly induced in BAL and serum of WT but not in ILC2^del^ mice after IL-33 treatment (Figure 6, E and F). In addition to stimulating eotaxin production, IL-4 and IL-13 are also known to activate endothelial cells to upregulate surface VCAM-1 (19, 20), which can mediate eosinophil arrest and migration (21). Indeed, IL-33–treated IL4Rα^–/–^ mice showed reduced vascular staining for VCAM- 1 (Supplemental Figure 7C) compared with controls. By comparison, PECAM-1 was minimally present and unchanged, and ICAM-1 was equally and ubiquitously expressed on epithelial cells, suggesting VCAM-1 upregulation as a mechanism through which IL-13 acts to potentiate eosinophil tissue ingress.

## Discussion

Here we suggest a role for ILC2s and IL4Rα signaling in the pathogenesis of EGPA and generate a mouse model for study of this rare but disabling disease. We find that patients with EGPA had increased levels of IL-25, TSLP, and sST2 in serum and had a decrease in circulating ILC2s. We present a mouse model of eosinophilic vasculitis that recapitulates key aspects of human EGPA, in which IL-33 induces ILC2- and IL4Rα/STAT6-dependent transformation of established hypereosinophilia into severe tissue inflammation.

Our finding of elevated IL-25, TSLP, and sST2 in patients with EGPA corroborates previous reports (12, 13). Though we could not reliably measure IL-33 in this study, we note that the interpretation of serum IL-33 levels is complicated even when technically feasible due to sequestration by serum sST2 (22) — a system most likely devised to constrain IL-33 activity to the tissue of origin to prevent immunopathology. Further technical advances will be needed to determine whether IL-33 is elevated in affected tissues of patients with EGPA. Whether these alarmins are cause or consequence of injury remains uncertain. Immunopathology per se (e.g., damage attributable to excessive eosinophil degranulation in tissue) could be the sole cause for release of these cytokines. However, the propensity of EGPA to develop late in life and its fluctuating activity suggest that environmental triggers may be important in disease pathogenesis (23). Thus, a reasonable hypothesis is that environmental insults provoke the initial release of cytokines such as IL-33 at the mucosal barrier and initiate transformation to the vasculitic phase in a previously hypereosinophilic host, which then propagates further alarmin release and immunopathology.

Our finding that ILC2s were decreased in blood of patients with EGPA also replicates other studies (24). A conflicting report compared patients with EGPA with patients with asthma, who likely have abnormal numbers of circulating ILC2s (13). Though a decrease in circulating ILC2s seems paradoxical, we point out that ILC2s are largely tissue-resident. Circulating cells are capable of populating peripheral tissue pools — which are largely regenerated by local progenitors — when turnover or demand is high (25–27). Thus, circulating ILC2s may be depleted during active EGPA because they are recruited to stressed or damaged tissues.

Though we and others found elevations in multiple tissue cytokines in patients, only IL-33 induced pulmonary inflammation consisting of a mixed cellular infiltrate, giant cells, pulmonary capillaritis, and eosinophilic necrosis in hypereosinophilic mice. Mouse lung ILC2s are relatively insensitive to IL-25 compared with IL-33 (28) due to low expression of the receptor (29). Although tissue-specific receptor expression and cytokine sensitivity in human ILC2s are not fully established (14, 30, 31), such data would inform cytokine candidacy for induction of EGPA in patients and reveal patient-specific correlations between individual tissue cytokine changes and specific organ involvement.

IL-33 has been reported to directly increase endothelial activation and vascular permeability (32, 33). However, vascular leak was significantly improved by removal of ILC2s or IL4Rα/STAT6 signaling in our system. Similarly, eosinophil extravasation was significantly inhibited by the loss of IL4Rα signaling, at least in part due to reduced induction of VCAM-1 and eotaxin. By our estimate, approximately 50% of the vascular leak and eosinophil migration after IL-33 treatment is ILC2 dependent and IL4Rα dependent, whereas the other 50% may be attributable to direct effects of IL-33 on the endothelial or epithelial cells, or other mechanisms. In light of their role in regulating pulmonary edema after IL-33 treatment, we find it notable that IL-33-responsive ILC2s reside in the structures that are most critical for clearing edema fluid in the lungs: the adventitial cuffs of bronchovascular bundles (34).

Because ILC2s produce IL-13 in the inflamed lung but minimal IL-4 (35), IL-13 is more likely drive the phenotype, even though both cytokines signal through IL4Rα. Within this paradigm, IL-33 may activate ILC2s to produce IL-13, which in turn stimulates eotaxin production from parenchymal cells. However, ILC2s have also been reported to produce eotaxin (36), and therefore it is possible that ILC2s themselves may be the source of this chemoattractant. Interestingly, we observed that although ILC2^del^ rescued mice from alveolar hemorrhage and BAL hypereosinophilia, histopathologic findings were further improved in mice entirely deficient in IL-4 and IL-13 signaling. This difference may be due to ILC2s that produce IL-13 but not IL-5 (therefore not deleted in ILC2^del^ mice) or other cell types that produce IL-13 or IL-4. Although the lack of protection from IL-33-induced pulmonary edema in Rag1^–/–^ mice argues against a central role for T cells in the acute vasculitic phase, Th2 cells may be critical for the development of the asthmatic and infiltrative phases, whereas ILC2s drive the vasculitic phase. Of note, although the ectopic expression of IL-5 in our mice precludes the ability to investigate ILC2s as a source of IL-5, this is a point for further investigation among patients with EGPA.

Rare cases of vasculitic syndromes consistent with EGPA are reported during treatment with dupilumab, as well as with placebo, in the context of reduced corticosteroid dose in patients with asthma (37, 38). We surmise that some cases of EGPA may have been misdiagnosed as simple eosinophilic asthma in these trials rather than IL4Rα blockade leading to vasculitis per se. Dupilumab is associated with increases in circulating eosinophils (39), which seems undesirable in a disease characterized by hypereosinophilia and is worthy of caution. However, because hypereosinophilia is insufficient to cause pathology without extravascular migration and activation, our findings raise the question of whether dual blockade of eosinophils *and* IL-33 or IL4Rα could be useful for therapy in EGPA. Indeed, because dupilumab decreases eotaxin expression in patients (39), augmented peripheral eosinophilia could be a direct consequence of blocking extravascular migration and activation.

In sum, using patient samples and mouse modeling, we suggest a mechanism for the transition from hypereosinophilia to vasculitis in EGPA. In this model, environmental insults or tissue damage led to the release of tissue cytokines such as IL-33. This may have stimulated ILC2s to produce IL-13, which increases pulmonary vascular permeability, eotaxin, and VCAM-1 expression, facilitating the migration of eosinophils into the extravascular space. Once in the extravascular space and in contact with activating tissue factors, these eosinophils degranulated and caused tissue damage, likely promoting further release of activating alarmins. Our data provide a useful mouse model for further study of this rare and complex disease and suggest that therapeutic targeting of IL-33, IL4Rα, and/or ILC2s may offer benefit in eosinophilic vasculitis.

## Methods

### Patients with EGPA and healthy controls.

Serum and PBMCs from healthy controls were obtained at the Etablissement Français du Sang, Saint-Antoine Hospital, Paris, France. Patients with EGPA, described in Tables 1 and 2, fulfilled the American College of Rheumatology criteria (40) and the 2012 Chapel Hill Consensus Conference definition (1). Patients were determined to be in the active phase of EGPA if they had a Birmingham vasculitis activity score (BVAS; version 3.0) of 3 or greater (41), or in remission if they had a BVAS of 0. Patients with EGPA were identified at the Vasculitis Reference Center for Vasculitis, Cochin Hospital, Paris, France, and patients with available frozen sera were selected for cytokines measurement. Samples from age- and sex-matched healthy controls were provided to the investigators in blinded fashion by the Etablissement Français du Sang without demographics included.

### Measurement of cytokines and ILC2s from patients.

PBMCs were purified from whole blood using Ficoll-Hypaque (GE Healthcare) and analyzed on a LSR Fortessa (BD Biosciences). Viability was assessed using LIVE/DEAD viability assay (Thermo Fisher Scientific). A gating strategy for ILC2s is shown in Supplemental Figure 1C. sST2, IL-25, TSLP, and IL-33 were quantitated using commercially available ELISAs from Tebu Bio, Peprotech, eBioscience, and R&D Systems, respectively. IL-5 and IL-10 were quantified by Luminex (Thermo Fisher Scientific) using the Th1/Th2/Th17 Magnetic 8-Plex Panel. Abs are listed in Supplemental Table 1.

### Mice.

IL5Tg (5), Arg1^YFP^ (42), *Il5*^Red5Cre^ (Red5) (10), ILC2-deleted (ILC2^del^) (10); STAT6^–/–^ (43), Smart13 (35), IL4Rα^–/–^ (44), and IL4^–/–^/IL13^–/–^ (45) mice have been described. Mice were derived or backcrossed on a C57BL/6J background for more than 10 generations, and housed under specific pathogen–free conditions with 12-hr light/day cycles and ad libitum access to food and water. Experiments were performed on age- and sex-matched male and female mice between 8 weeks and 16 weeks of age, except for the eotaxin experiments, in which mice were 6 months of age. IL-33 (BioLegend or R&D), TSLP (eBioscience), or IL-25 (R&D) were administered intranasally at 0.5 μg per dose, 3 times a week for 2 weeks, and mice were analyzed 1 day after the final dose. For intravascular labeling experiments, mice were injected intravenously with 3 μg of anti-mouse CD45 Ab (clone 30-F11) 3 minutes prior to sacrifice. Eotaxin was measured from serum using the Mouse CCL11/Eotaxin Quantikine ELISA Kit (R&D).

### Mouse cell isolation and flow cytometry.

PBMCs were isolated using Histopaque-1083 (Sigma). BAL was obtained by instilling 1.5 mL of saline via tracheal cannulation, followed by saline perfusion. Lung single cell suspensions were obtained by tissue digestion with 50 μg/mL Liberase (Roche) and 25 μg/mL DNAse I (Roche). Flow cytometry was performed on an LSR Fortessa and analyzed using FlowJo (Tree Star). Dead cells were excluded by LIVE/DEAD or DAPI. A representative gating strategy for eosinophils and ILC2s is shown in Supplemental Figure 2. For IL-13 assessment by Smart13 as in Figure 3, lung ILC2s were gated as lineage-negative, Thy1.2^+^, CD3^–^, Arg1^YFP+^, Red5^+^. Abs are listed in Supplemental Table 1.

### Microscopy.

Four percent paraformaldehyde-fixed tissues were embedded in paraffin for H&E staining or OCT (Sakura) for immunofluorescence and imaged on a Leica light microscope (H&E) or Nikon A1R confocal microscope. Histologic appearance was evaluated in a blinded manner at a uniform anatomic position, and a severity score of 0 (none) to 3 (severe) was assigned for alveolitis, inflammatory infiltrates, and giant cells. Capillaritis was defined as presence (score, 1) or absence (score, 0) of hemorrhage associated with perivascular infiltration of macrophages, neutrophils, lymphocytes, and/or eosinophils. If perivascular infiltration was present without overt hemorrhage, images were scored as 0.5.

### Lung physiology.

Mice were anesthetized with ketamine and xylazine and intubated with an 18-gauge cannula for analysis on the FlexiVent FX NPFE and Aeroneb system (SciReq). Pancuronium was used to paralyze mice before studies of lung mechanics and methacholine challenges at the indicated doses. ELW and EVPE were measured as previously described (46).

### Statistics.

Analyses were performed with GraphPad Prism 8 using 2-tailed nonparametric Mann-Whitney *U* test or 1-way ANOVA with post hoc testing as appropriate. Data shown in the figures represent mean ± SEM.

### Study approval.

For human studies, all subjects provided written informed consent according to the Declaration of Helsinki (CPP authorization number 2011-12767). Protocols were approved by the INSERM IRB (NEUTROVASC DR-2012-002) and the Hospital Cochin Ethics Committee. All procedures on mice were approved by the UCSF Animal Care and Use Committee.

## Author contributions

MEK and BT designed and performed experiments, interpreted data, contributed to discussion, and wrote the manuscript. RML designed experiments, interpreted data, contributed to discussion, and edited the manuscript. SVD designed experiments and contributed to discussion. RRRG, JD, CJD, CT, and LM performed experiments and interpreted data.

## Supplementary Material

Supplemental data

## Figures and Tables

**Table 2:**
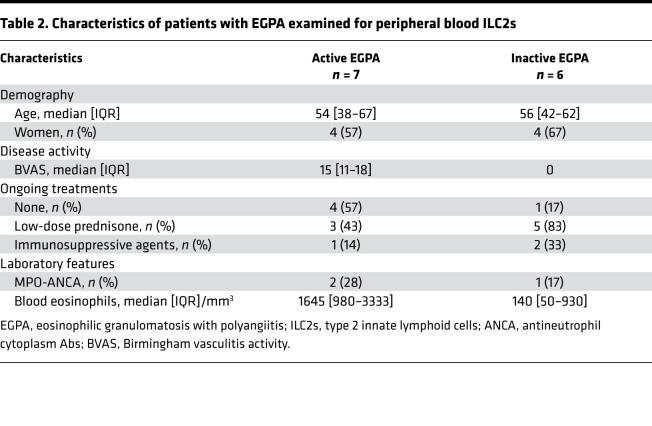
Characteristics of patients with EGPA examined for peripheral blood ILC2s

**Table 1:**
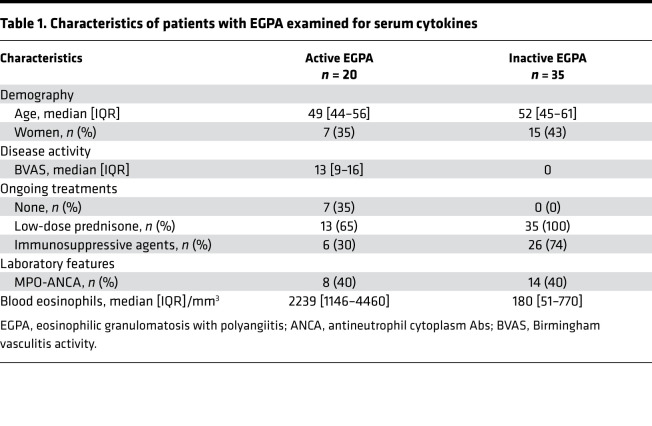
Characteristics of patients with EGPA examined for serum cytokines

## Version 1 05/11/2021

In-Press Preview

## Version 2 06/22/2021

Electronic publication
