# Supplementary material for: A role for IL-33–activated ILC2s in eosinophilic vasculitis
Source: JCI Insight. 2021 Jun 22;6(12):e143366. doi: 10.1172/jci.insight.143366 (PMC8262498; doi:10.1172/jci.insight.143366)
Supplement: Supplemental data [file jciinsight-6-143366-s058.pdf]

Supplemental Materials

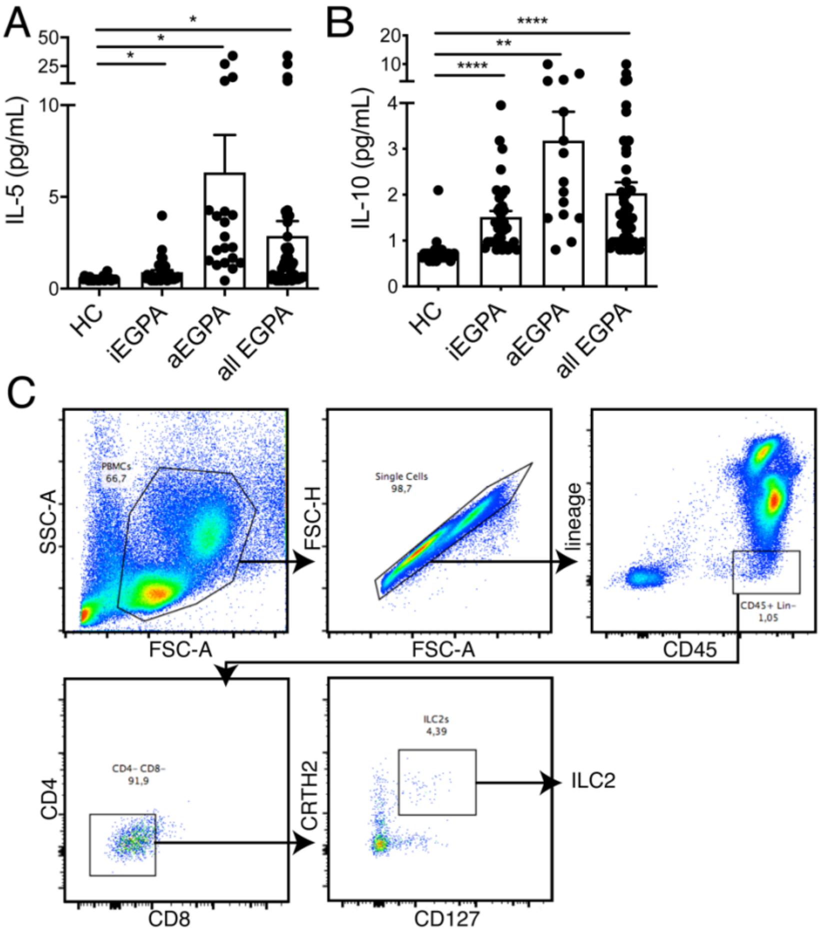

Supplemental Figure 1: Human patients with EGPA have evidence of type 2 inflammation.

(A) Serum IL-5 and **(B)** IL-10 from healthy controls (HC) or EGPA patients with inactive (iEGPA) or active (aEGPA) disease. n=20-53 per group. ****p<0.0001; ***p<0.001; **p<0.01; *p<0.05; ns = non-significant by Brown-Forsythe and Welch ANOVA with Dunnett post-hoc testing. p=0.06 and 0.08 for IL-5 and IL-10, respectively, for the difference between active and inactive disease. **(C)** Representative flow cytometry gating strategy for ILC2s in human patients.

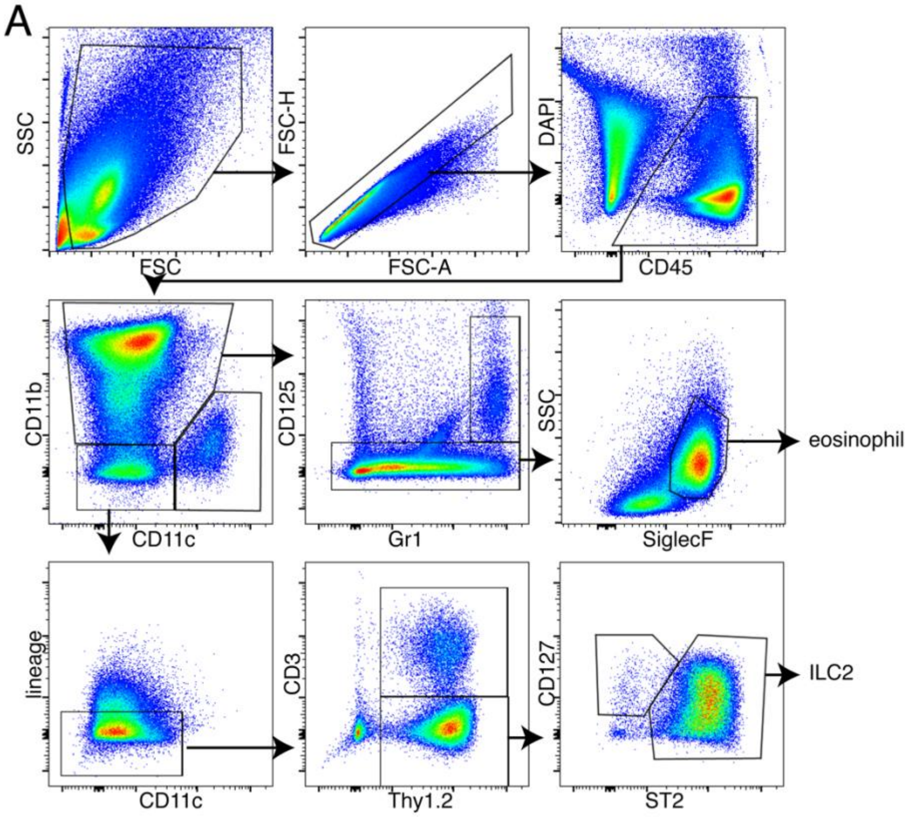

Supplemental Figure 2: Gating strategy for ILC2s.

(A) Representative flow cytometry gating strategy for eosinophils and ILC2s in mice.

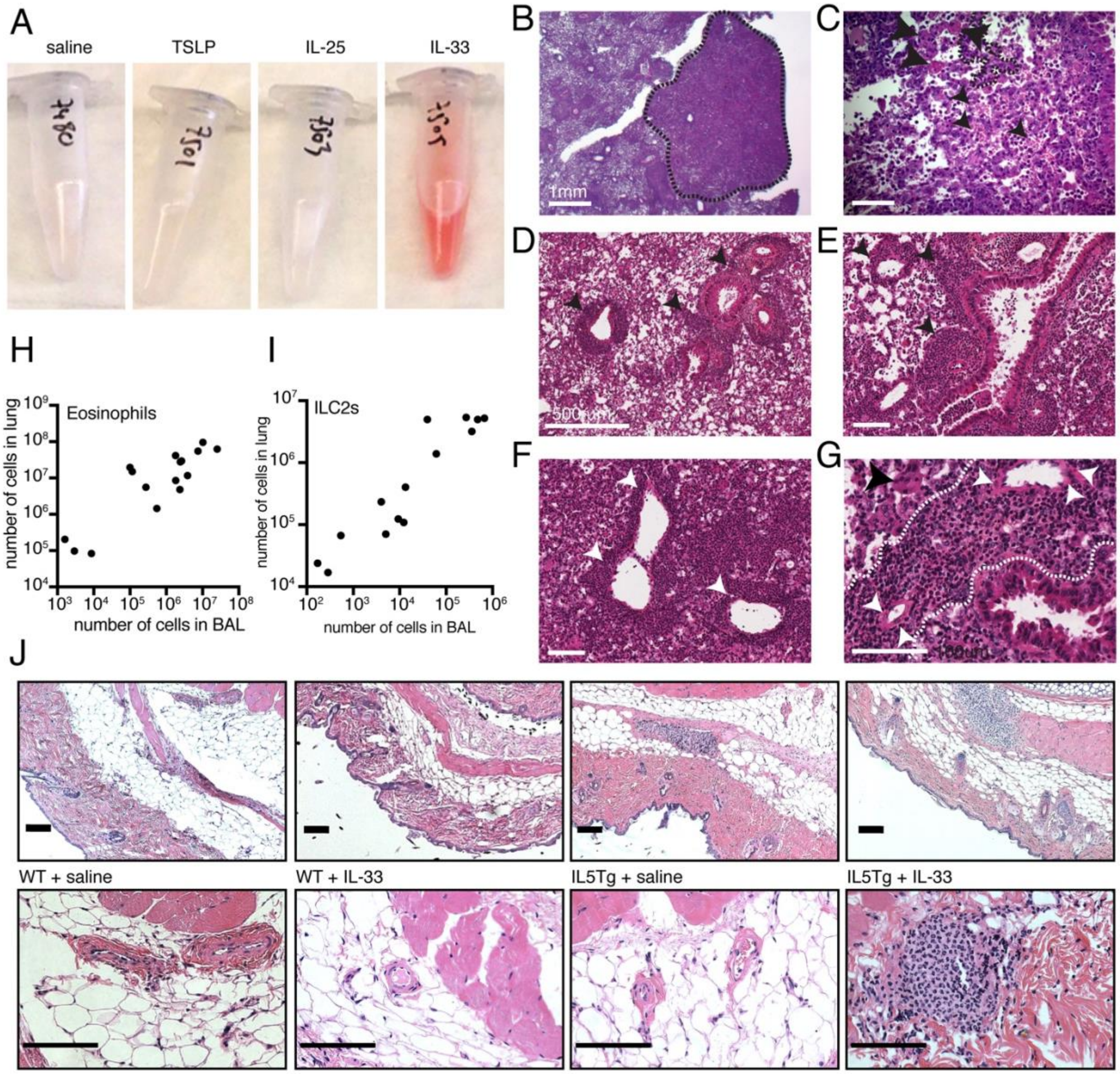

Supplemental Figure 3: IL-33 induces vasculitis in hypereosinophilic mice.

(A) Representative BAL from saline-, TSLP-, IL-25, or IL-33-treated IL5Tg mice. Lungs of IL-33-treated IL5Tg mice at various magnification showing **(B)** hemorrhage and diffuse alveolar filling **(C)** giant cells (arrowheads), alveolar wall infiltration (dotted outlines) and hemorrhage (arrows) **(D-E)** peribronchial and perivascular clusters **(F-G)** eosinophilic infiltrates (dotted lines), eosinophil invasion of the vessel walls (white arrowheads), and giant cells (black arrowhead).

Scale bars represent 100 μm unless otherwise specified. **(H)** Correlation between numbers of eosinophils among saline- or IL-33-treated WT or IL5Tg mice. n=16 mice. Pearson r = 0.8718 for correlation between log values. **(I)** Numbers of ILC2s in lung and BAL among saline- or IL-33-treated WT or IL5Tg+ mice. n=14 mice. Pearson r = 0.9354 for correlation between log values. **(J)** H&E staining of skin samples from IL-33-treated WT or IL5Tg mice. Representative of ≥3 mice/group. 10x (top row) or 40x (bottom row) magnification. Scale bars represent 100 μm.

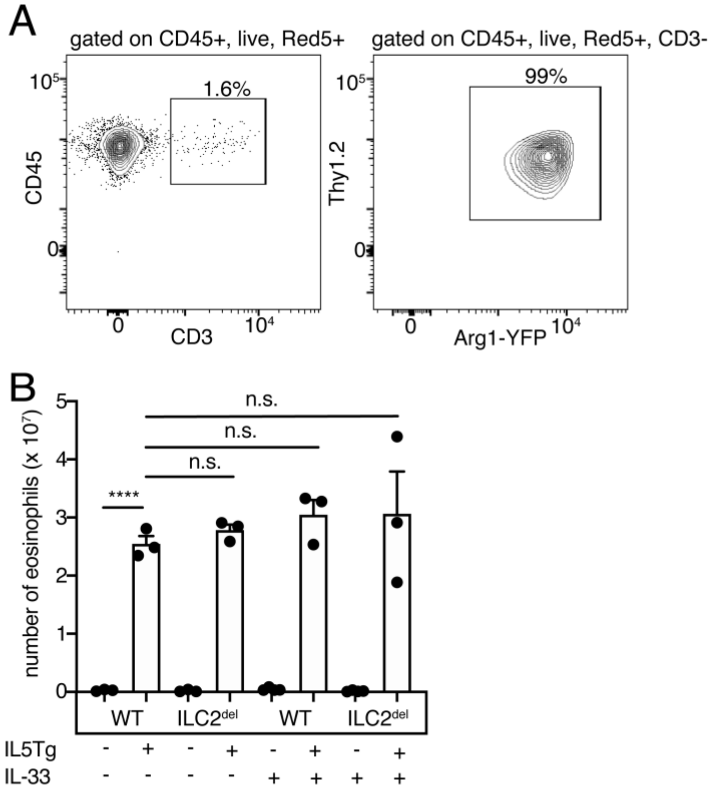

Supplemental Figure 4: ILC2s are the major source of IL-5 that are deleted in ILC2^{del} mice.

(A) Representative flow cytometry plots of total CD45+ Red5+ cells demonstrated that even after IL-33 treatment, only ~1-2% of IL-5+ (Red5+) cells were CD3+, and the remaining Red5+ cells were Thy1.2+, Arg1^{YFP}+ ILC2s. **(B)** Peripheral blood eosinophils are equivalently elevated in IL5Tg mice on ILC2^{del} and WT backgrounds. Error bars indicate SEM. ****$p<0.0001$ by 1-way ANOVA with Sidak post-hoc testing. n=3-4 per group.

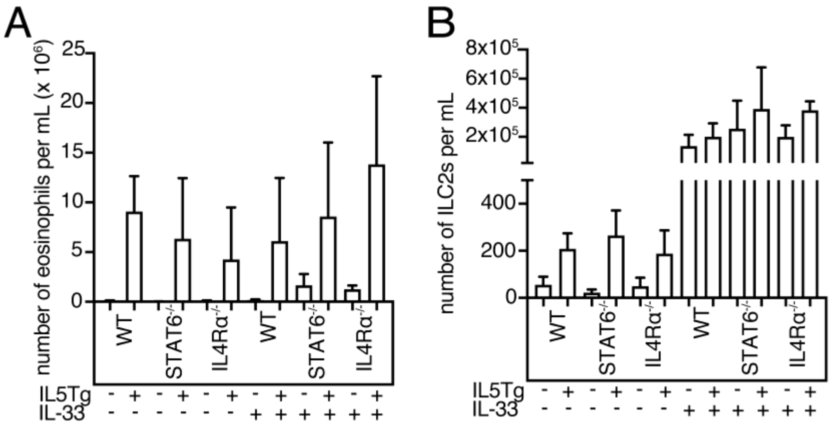

Supplemental Figure 5: Loss of IL-13 signaling protects from eosinophilia and alveolar hemorrhage without altering total peripheral eosinophils or ILC2s.

(A) Peripheral blood hypereosinophilia is present in all IL5Tg mice and unaffected by $STAT6^{-/-}$ or $IL4R\alpha^{-/-}$. n= 2-4 mice/ group. **(B)** IL-33 induces blood ILC2s equally in WT, $STAT6^{-/-}$ and $IL4R\alpha^{-/-}$. n= 2-4 mice/group.

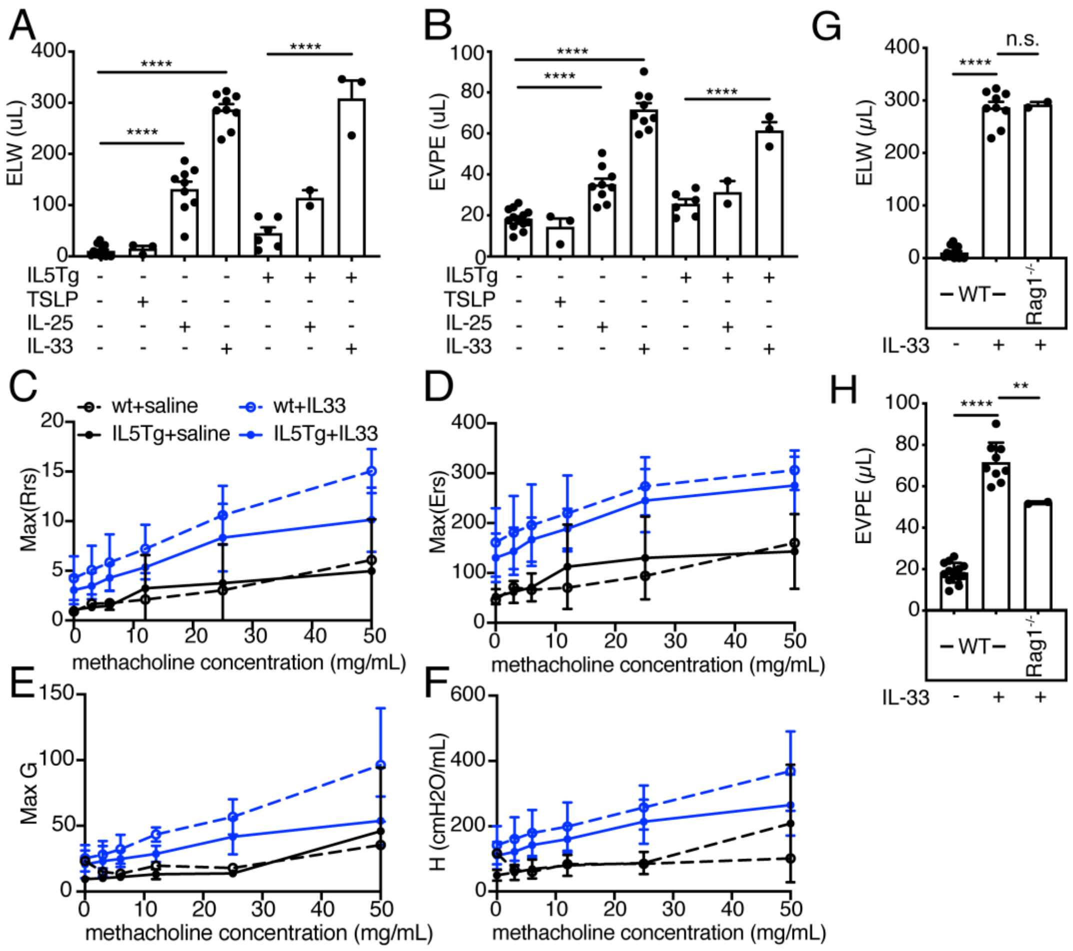

Supplemental Figure 6: IL-33 induces inflammatory pulmonary edema not seen with other alarmins, but does not result in bronchoreactivity.

(A) Excess/extravascular lung water in saline-, TSLP-, IL-25- or IL-33-treated WT or IL5Tg+ mice. Error bars indicate SEM. ****p<0.0001 by 1-way ANOVA with Sidak post-hoc testing. n=3-13 per group. **(B)** Extravascular pulmonary equivalents (EVPE) measured from saline-, TSLP-, IL-25- or IL-33-treated WT or IL5Tg+ mice. Error bars indicate SEM. ****p<0.0001 by 1-way ANOVA with Sidak post-hoc testing. n=3-13 per group. **(C-F)**. Respiratory system resistance (“Rrs”), respiratory system elastance (“Ers”), tissue damping (“G”), and tissue elastance (“H”), respectively, of saline- or IL-33-treated WT or IL5Tg mice during methacholine challenge. n=6-8 per group. **(G)** Excess/extravascular lung water or **(H)** extravascular pulmonary equivalents

measured from saline- or IL-33-treated WT or Rag1$^{-/-}$ mice. n=2-13 per group. ****p<0.0001; ***p<0.001; **p<0.01; *p<0.05; ns = non-significant by ANOVA with Dunnett post-hoc testing.

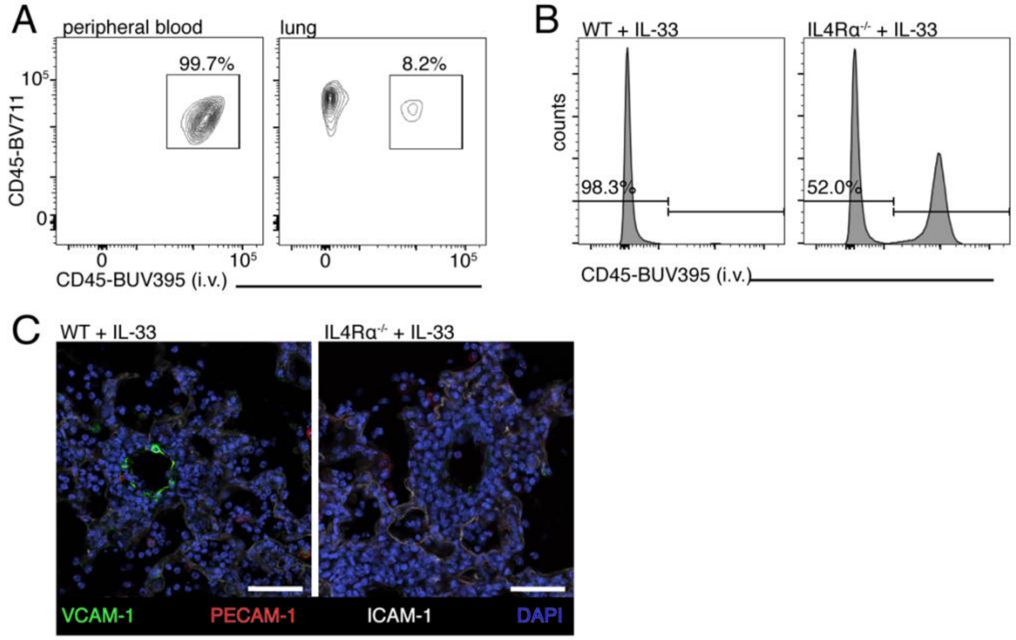

<u>Supplemental Figure 7: Intravenous labelling demonstrates that IL4Rα signaling is required for eosinophils to enter lung parenchyma after IL-33 treatment.</u>

(A) Representative flow cytometry plots of peripheral blood (left panel) and lung (right panel) total CD45+ cells (CD45-BV711) from the same IL-33-treated WT mouse. In blood, nearly 100% of peripheral blood CD45+ cells were labelled after i.v. injection of anti-CD45-BUV395 antibody, whereas the majority of CD45+ lung cells were not labelled by i.v. injection, reflecting extravascular positioning. **(B)** Representative histograms of intravascularly labelled (CD45-BUV395+) and extravascular/unlabeled (CD45-BUV395-) eosinophils in IL-33-treated IL4R$\alpha^{-/-}$ or WT mice. Pre-gated on single, live, CD45+, CD11b+, CD125-, SiglecF+, SSC-hi cells. (**C**) Representative images from WT (left panel) or IL4R$\alpha^{-/-}$ (right panel) mice treated with IL-33. VCAM-1 is stained in green, PECAM-1 in red, ICAM-1 in white, nuclei (DAPI) in blue. Scale bars represent 50 μm.

Supplemental Table 1: antibodies used

Antigen	Clone	vendor	Catalog #
Analysis of mouse cells/tissues			
**Anti-Mouse FACS antibodies**			
Anti-mouse CD45 BUV395	30-F11	BD	Cat# 565967
Anti-mouse CD45 BV 711	30-F11	BioLegend	Cat# 103147
Anti-mouse CD125 PE	T21	BD Biosciences	Cat# 558488
Anti-mouse SiglecF PerCP-Cy™5.5	E50-2440	BD Biosciences	Cat# 565526
Anti-mouse CD11b BV650	M1/70	BioLegend	Cat# 101239
Anti-mouse CD11c BV785	N418	BioLegend	Cat# 117335
Anti-mouse CD127 APC-eFluor® 780	A7R34	eBioscience/ThermoFisher	Cat# 47-1271-82
Anti-mouse Gr-1 APC	RB6-8C5	BioLegend	Cat# 108412
Anti-mouse ST2 PE	DJ8	MD Biosciences	Cat# 101001PE
Anti-mouse ST2 Biotin	DJ8	MD Biosciences	Cat# 101001B
Anti-mouse CD3 PE-Cy7	17A2	BioLegend	Cat# 100220
Anti-mouse CD3 APC-Cy7	17A2	BioLegend	Cat# 100222
Anti-mouse CD3 BV711	17A2	BioLegend	Cat# 100241
Anti-mouse Thy1.2 BV605	53-2.1	BioLegend	Cat# 140317
Anti-mouse Thy1.2 BV785	30-H12	BioLegend	Cat# 105331
Anti-mouse CD4 PerCP	RM4-5	eBioscience/ThermoFisher	Cat# 45-0042-82
Anti-human/mouse GATA3 AF488	TWAJ	eBioscience/ThermoFisher	Cat# 53-9966-42
Anti-mouse Foxp3 PE	FJK-16s	eBioscience/ThermoFisher	Cat# 12-5773-82
Anti-mouse Tbet PE-Cy7	4B10	eBioscience/ThermoFisher	Cat# 25-5825-80
**Anti-Mouse Lineage cocktail**			
Anti-mouse CD8α PB	53-6.7	BioLegend	Cat# 100725
Anti-mouse CD49b PB	DX5	BioLegend	Cat# 108918
Anti-mouse NK-1.1 PB	PK136	BioLegend	Cat# 108722

Anti-mouse Gr-1 PB	RB6-8C5	BioLegend	Cat# 108430
Anti-mouse TER-119 PB	TER-119	BioLegend	Cat# 116232
Anti-mouse FcεRIa PB	MAR-1	BioLegend	Cat# 134314
Anti-mouse CD3 PB	17A2	BioLegend	Cat# 100214
Anti-mouse CD19 PB	6D5	BioLegend	Cat# 115523
Anti-mouse CD11c PB	N418	BioLegend	Cat# 117322
Anti-mouse/human CD11b PB	M1/70	BioLegend	Cat# 101224
Anti-mouse CD4 PB	RM4-5	BioLegend	Cat# 100428
Anti-mouse F4/80 PB	BM8	BioLegend	Cat# 123124
**Anti-human FACS antibodies**			
Anti-human CD4 APC	RPA-T4	eBioscience	Cat# 17-0049
**Immunofluorescence**			
Anti-mouse VCAM-1 AF488	421	BioLegend	Cat# 105701
Anti-mouse CD31 (PECAM1)	MEC 13.3	BD Biosciences	Cat# 553370
Anti-mouse ICAM-1 AF647	YN1/1.7.4	BioLegend	Cat# 116113
Goat anti-rat IgG AF555		Invitrogen	Cat# A21434
Analysis of human cells/tissues			
**Anti-Human FACS antibodies**			
Anti-human CD45 APC	HI30	Ozyme/BioLegend	Cat# BLE304012
Anti-human CD127/IL7-RA PE	A019D5	Ozyme/BioLegend	Cat# BLE351304
Anti-human CD294 BV421	BM16	Ozyme/BioLegend	Cat# BLE350112
Anti-human CD8α PerCP-Cy™5.5	SK1	Ozyme/BioLegend	Cat# BLE344710
Anti-human CD4 BV605	RPA-T4	Ozyme/BioLegend	Cat# BLE300556
**Anti-Human Lineage cocktail**			
Anti-human CD3 FITC	HIT3a	Ozyme/BioLegend	Cat# BLE300306
Anti-human CD11c FITC	3.9	Ozyme/BioLegend	Cat# BLE301604
Anti-human CD14 FITC	M5E2	Ozyme/BioLegend	Cat# BLE301804

Anti-human CD19 FITC	HIB19	Ozyme/BioLegend	Cat# BLE302206
Anti-human CD20 FITC	2H7	Ozyme/BioLegend	Cat# BLE302304
Anti-human CD56 FITC	HCD56	Ozyme/BioLegend	Cat# BLE318304
Anti-human CD123 FITC	6H6	Ozyme/BioLegend	Cat# BLE306014
Anti-human FcεR1 AF488	AER-37 (CRA-1)	Ozyme/BioLegend	Cat# BLE334640
**Anti-human ELISA**			
Human Th1/Th2/Th17 Magnetic 8-Plex Panel		Life Technologies	LHC0015M
IL-17E/IL-25		Peprotech	900-M234
sST2		R&D	DST200
TSLP		eBioscience	88-7497-86
